# Quality of Life in Patients with Ureteral Stones: Translation and Validation of the Brazilian Version of the Cambridge Ureteral Stone PROM (Br-CUSP)

**DOI:** 10.1590/S1677-5538.IBJU.2024.0551

**Published:** 2025-11-30

**Authors:** Alexandre Danilovic, Daniel Gabriele Sucupira, Oliver Wiseman, Fabio Cesar Miranda Torricelli, Giovanni Scala Marchini, Carlos Batagello, Rodrigo Perrela, Fabio Carvalho Vicentini, William C. Nahas, Eduardo Mazzucchi

**Affiliations:** 1 Universidade de São Paulo Faculdade de Medicina Hospital das Clínicas São Paulo SP Brasil Departamento de Urologia, Hospital das Clínicas - HCFMUSP, Faculdade de Medicina, Universidade de São Paulo, São Paulo, SP, Brasil; 2 Cambridge University Addenbrooke's Hospital Department of Urology Cambridge United Kingdom Department of Urology, Addenbrooke's Hospital, Cambridge University, Cambridge, United Kingdom

**Keywords:** Quality of Life, Ureteral Calculi, Patients

## Abstract

**Purpose:**

There is currently no validated instrument in Brazil specifically designed to assess the quality of life (QoL) of patients with ureteral stones. The Cambridge Ureteral Stone Patient-Reported Outcome Measure (CUSP) is a self-administered questionnaire that evaluates the QoL impact of ureteral stones over the preceding seven days. This study aimed to translate, culturally adapt, and validate the CUSP for Brazilian Portuguese (Br-CUSP) for clinical and research applications.

**Materials and Methods:**

The CUSP questionnaire was translated into Portuguese according to Guillemin's cross-cultural adaption guidelines. Patients with and without ureterolithiasis completed both the Br-CUSP and SF-12 questionnaires. Psychometric validation included assessment of internal consistency, test-retest reliability, convergent validity, and discriminant validity.

**Results:**

A total of 156 participants completed both questionnaires. No inconsistencies emerged during univariate analysis. Confirmatory factor analysis supported the six-factor model with satisfactory fit indices. All factor loadings exceeded 0.50. Internal consistency was high across all domains (Cronbach's α = 0.72 - 0.98; McDonald's ω = 0.73 - 0.98). Test-retest reliability demonstrated strong temporal stability. Inter-domain correlations (Spearman's p = 0.45 - 0.82) supported structural coherence. Convergent validity was confirmed through inverse correlations with SF-12 scores. Discriminant validity was demonstrated by significant score differences between patients with and without ureteral stone, with large effect sizes.

**Conclusions:**

The Brazilian Cambridge Ureteral Stone Patient-Reported Outcome Measure is a valid, reliable tool for assessing health-related quality of life in Brazilian patients with ureteral stones. Its implementation can enhance both clinical assessment and research into patient-centered outcomes in urolithiasis.

## INTRODUCTION

Urolithiasis is a highly prevalent condition that significantly impairs patients’ quality of life (QoL) due to unexpected pain, discomfort, and temporary disability (1-5). Recurrence rates range from 30% to 50% within five years, imposing a substantial and often recurrent burden on patients’ daily lives (6-10).

Despite its clinical impact, outcome measures primarily focus on stone-free rates (SFR), neglecting patient-centered outcomes such as QoL (11). Notably, neither the European Association of Urology nor the American Urological Association guidelines currently recommend the routine incorporation of QoL metrics in treatment planning for ureteral stones (12, 13).

Integrating health-related quality of life (HRQoL) assessment into clinical care offers a more holistic view of disease burden by capturing patient's physical, psychological, and social functioning. This approach aligns with patient-centered care principles by ensuring treatment strategies reflecting both clinical efficacy and individual patient experiences (14, 15).

Patient-reported outcome measures (PROMs) are validated instruments designed to objectively quantify the patient's perception of disease impact (16). The Cambridge Ureteral Stone PROM (CUSP) is a disease-specific, self-administered questionnaire comprising 26 items across six domains: pain, fatigue, daily activities, sleep disturbances, anxiety, and urinary symptoms. Each item is rated on a five-point Likert scale, with higher scores indicating worse HRQoL (17). Unlike other HRQoL tools, the CUSP is specifically designed for ureteral stone patients and uniquely evaluates symptom burden over the preceding seven days, enhancing its clinical relevance for monitoring short-term treatment outcomes (12).

No validated instruments currently exist in Brazilian Portuguese to assess QoL specifically in patients with ureteral stones. We hypothesize that the CUSP questionnaire can be effectively validated for use in Brazil. Therefore, the objective of this study was to conduct a cross-cultural adaptation and psychometric validation of the CUSP questionnaire for Brazilian Portuguese (Br-CUSP), ensuring linguistic and conceptual equivalence while maintaining its measurement properties.

## MATERIALS AND METHODS

### Study Design and Participants

This prospective study was conducted at a specialized public university hospital between December 2022 and May 2023. Eligible participants were adults over 18 years old, fluent in Portuguese, with or without tomography verified ureteral stones. All participants provided written informed consent prior to enrollment. The study adhered to the principles of the Declaration of Helsinki and received ethical approval from the institutional review board (IRB approval number 64388822.9.0000.0068).

Exclusion criteria included the presence of kidney stones, other urological conditions, pelvic pain syndrome, use of anticholinergics, alpha-blockers, calcium channel blockers, phosphodiesterase type 5 inhibitors, age under 18 years, illiteracy, or known psychiatric disorder.

### Translation and Cultural Adaptation

The CUSP questionnaire was translated into Brazilian Portuguese by two independent native Portuguese-speaking translators with expertise in Urology. Next, a consensus meeting involving the authors was held. Subsequently, an independent bilingual professional back-translated the questionnaire into English. The original author compared both versions, resolving discrepancies through further consensus meetings. A pilot test was conducted with 20 patients to evaluate comprehension and clarity.

### Data Collection

Patients completed the self-administered Br-CUSP questionnaire twice, with a two to three hours interval between administrations to assess test-retest reliability. Discriminant validity was assessed using SF-12 Health Survey (version 1.0), a generic measure of health-related quality of life already translated and validated for Brazilian Portuguese (18). The SF-12 consists of two components: Physical Component Score (PCS-12) and Mental Component Score (MCS-12), with higher scores indicating better QoL. These scores are interpreted inversely relative to CUSP, in which higher scores denote worse HRQoL.

## Statistical Analysis

### Internal Structure Validity

Analyses were performed using JASP software (version 0.18.3). Confirmatory Factor Analysis (CFA) was conducted to assess the internal structure of the Br-CUSP, following the six-domain model originally proposed by Tran et al. (17). Given the categorical nature of the Likert-scale data, the mean- and variance-adjusted weighted least squares (WLSMV) estimator with robust standard errors based on polychoric correlations was used. A factor loading threshold of > 0.40 was applied.

Model fit was assessed using chi-square (X^2^), degrees of freedom (df), X^2^/df ratio (acceptable < 5; ideal < 3), Root Mean Square Error of Approximation (RMSEA; acceptable < 0.08), Comparative Fit Index (CFI; > 0.95), Tucker-Lewis Index (TLI; > 0.95), and Standard Root Mean Square Residual (SRMR; < 0.08).

### Internal Consistency and Reliability

Internal consistency of the Br-CUSP domains was assessed using Cronbach's alpha and McDonald's omega, with values ≥ 0.70 considered acceptable. Additionally, the Average Variance Extracted (AVE) was calculated to evaluate the proportion of variance captured by each construct relative to error variance, with AVE ≥ .50 considered adequate.

### Convergent Validity

Convergent validity was evaluated using Spearman's correlation (rho) between the Br-CUSP domains and SF12 scores. Negative correlations were expected, as higher Br-CUSP scores reflect worse HRQoL, while higher SF-12 scores reflect better HRQoL. Correlation values were interpreted as follows: ± 0.1 represents a small effect, ± 0.3 a medium effect, and ± 0.5 a large effect.

### Discriminant Validity

Discriminant validity was assessed by comparing Br-CUSP scores between patients with and without ureteral stones. Independent sample t-tests were used for comparisons. Levene's test assessed variance homogeneity, and Welch's statistic was used when homogeneity was not met. Bootstrapping procedures (1,000 resamplings; 95% CI BCa) corrected distribution normality deviations and increased result reliability (19). Effect sizes was calculated using Hedges’ g to adjust for unbalanced sample bias. Effect sizes were interpreted as follows: negligible effect (< 0.20); small effect (0.21- 0.39); medium effect (0.40 - 0.79); large effect (≥ 0.80).

## RESULTS

### Participants

Demographic and clinical characteristics are summarized in Table-1. A total of 156 patients completed both self-administered questionnaires. The sample was gender-balanced, comprising 78 males and 78 females (50.0%). The most common education level was high school (n = 72; 46.15%).

**Table 1:** Demographic and clinical features of the study population.

Feature		N	%
**Sex**			
	Female	78	50.0
**Education**			
	Incomplete	10	6.4
	Elementary school	36	23.1
	High school	72	46.2
	University graduate	26	16.7
	Postgraduate studies	12	7.7
**Race**			
	White	74	47.4
	Black / African American	19	12.2
	Asian	6	3.9
	More than one race	54	34.6
	Missing	3	1.9
**Occupation**			
	Student	7	4.5
	Working	100	64.1
	Unemployed	9	5.8
	Retired	23	14.7
	Housewife	17	10.9
**Ureteral stone**		129	82.7
**Previous stone event**		90	57.7
**Indwelling ureteral stent**		42	26.9
**Comorbidity**		67	43.0
**ASA**			
	I	77	49.4
	II	73	46.8
	III	5	3.2

ASA = American Society of Anesthesiologists Physical Status

Among the study cohort, 129 participants (82.7%) had ureteral stones confirmed by computed tomography, while 27 (17.3%) had no urinary stones. Of those with ureteral stones, 48 (30.7%) had stones located in the proximal ureter, and 42 (26.9%) had an indwelling double-J stent. A history of previous stone events was reported by 90 patients (57.7%). Most participants had no comorbidities (n = 89, 57.1%).

### Construct Validity

No univariate inconsistency was detected. Item means ranged from 1.92 to 3.25, with acceptable skewness and kurtosis values. The Kaiser-Meyer-Olkin (KMO) measure of sampling adequacy was 0.95, and Bartlett's test of sphericity was significant (X^2^ = 1675.1, df = 325; p < 0.001).

The results from the confirmatory factor analysis are presented in Supplementary material 1. All factor loadings were statistically significant and exceeded 0.50. The values indicate an adequate fit for the six-factor model (X^2^ = 180.855, df = 284, p < 0.001; X^2^/df ratio = 0.64, CFI =0 .99, TLI = 0.99, RMSEA = 0.05 [90% CI: 0.04 - 0.06], SRMR = 0.04).

### Internal Consistency

Internal consistency of the Br-CUSP domains was high across all six factors: Factor 1 - pain: α = 0.98 (95% CI: 0.97-0.98), ω = 0.98 (95% CI: 0.97-0.98); Factor 2 - fatigue: α = 0.95 (95% CI: 0.94-0.97), ω = 0.96 (95% CI: 0.94-0.97); Factor 3 - work, daily activities, and travel: α = 0.95 (95% CI: 0.93-0.96), ω = 0.95 (95% CI: 0.93-0.96); Factor 4 - sleep disturbances: α= 0.92 (95% CI: 0.89-0.94), ω= 0.92 (95% CI: 0.90-0.94); Factor 5 - anxiety: α = 0.89 (95% CI: 0.85-0.92), ω= 0.89 (95% CI: 0.84-0.93); Factor 6 - urinary symptoms: α = 0.72 (95% CI: 0.64-0.79), ω= 0.73 (95% CI: 0.63-.81). The Average Variance Extracted (AVE) was 0.90 for Factor 1, 0.88 for Factor 2, 0.90 for Factor 3, 0.76 for Factor 4, 0.79 for Factor 5, and 0.57 for Factor 6, all of which considered adequate.

### Inter-Domain Correlations

Supplementary material 2 summarizes the Spearman's correlation coefficient between Br-CUSP domains ranged from 0.45 to 0.82, indicating that each evaluated domain captures a distinct but related dimension of the patient's experience.

### Convergent Validity

Spearman's correlation coefficients between Br-CUSP domains and the two components of the SF-12 scale were significant and negative, as hypothesized. Correlations with the PCS-12 and MCS-12 scores ranged from −0.67 to −0.42, confirming that higher Br-CUSP scores were associated with lower SF-12 scores (Supplementary material 2).

### Discriminant Validity - Known Groups Analysis

Welch's t-test detected significant differences in all Br-CUSP domains between patients with and without ureteral stones (Table-2). In all comparisons, scores were higher (worse QoL) for the ureteral stone group, with large effect sizes ([Total score: ΔM = −51.72, 95% CI Bca (-56.17; −47.35), g = 3.10; Pain: ΔM = −19.39, 95% CI Bca (-21.24; −17.56), g = 2.87; Fatigue: ΔM = −9.59, 95% CI Bca (-10.70; −8.44), g = 2.30; Work: ΔM = −6.19, 95% CI Bca (-6.97; −5.40), g = 2.32; Sleep: ΔM = −7.79, 95% CI Bca (-8.79; −6.73), g = 2.39; Anxiety: ΔM = −5.62, 95% CI Bca (-5.62; −3.80), g = 1.76; Urinary Symptoms: ΔM = −3.99, 95% CI Bca (-4.65; −3.37), g = 1.76]).

**Table 2:** Evidence of discriminant validity.

Domain	Patients without ureteral stone (n = 27)	Patients with ureteral stone (n = 129)	Welch	Df	Difference	CI 95% - Bca Bootsstrap (Lower; Upper)		Effect's size (Hedges'g)
**Total Score**	**30.93 (4.27)**	**82.64 (23.11)**	-23.57*	**153.11**	**-51.72**	**-56.17**	**-47.35**	**3.10**
Pain	8.52 (1.34)	27.91 (9.42)	-22.33*	147.20	-19.39	-21.24	-17.56	2.87
Fatigue	5.78 (1.22)	15.37 (5.75)	-17.19*	153.93	-9.59	-10.70	-8.44	2.30
Work	3.74 (1.29)	9.93 (3.53)	-15.56*	114.58	-6.19	-6.97	-5.40	2.32
Sleep	5.56 (1.91)	13.34 (4.18)	-14.98*	86.65	-7.79	-8.79	-6.73	2.39
Anxiety	3.96 (1.85)	8.74 (3.35)	-10.33*	67.45	-4.77	-5.62	-3.80	1.76
Urinary Symptoms	3.37 (0.74)	7.36 (3.10)	-12.94*	151.72	-3.99	-4.65	-3.37	1.76

* p <0 .001

Df = degrees of freedom

Welch = Welch's t-value from independent-samples t-test allowing unequal variances. Negative values indicate higher symptom scores in patients with ureteral stones compared with healthy participants

### Test-Retest Reliability

Spearman's correlations for the CUSP-Br domains between time 1 (baseline) and time 2 were high (rho 0.96 – 0.99), indicating excellent temporal stability.

## DISCUSSION

This study presents the first cross-cultural adaptation and validation of a disease-specific QoL questionnaire for patients with ureteral stones into Brazilian Portuguese (Br-CUSP), while preserving its psychometric robustness. Understanding patient's subjective experiences and emotional burden is critical in ureteral stone disease, as it directly informs clinical management and enhances patient-centered care. By offering a comprehensive, symptom-focused assessment, Br-CUSP serves as a robust patient-reported outcome measure that captures the unique and acute burden of ureteral stone disease on patients’ daily lives. Furthermore, the Br-CUSP enables standardized, culturally relevant assessment of QoL, facilitating comparative studies, epidemiological research, and clinical trials tailored to the Brazilian population. This tool supports the development of evidence-based interventions, improves understanding of disease burden across diverse socioeconomic and regional contexts, and fosters international collaboration by aligning Brazilian urological research with global standards.

Our findings confirm that Br-CUSP is a reliable and valid instrument for evaluating health-related quality of life in this patient population. Reliability of Br-CUSP was demonstrated by high internal consistency across all domains and test-retest strong correlation demonstrated temporal stability. The two to three hours retest interval is appropriate given the acute nature of ureteral colic symptoms and the questionnaire's seven-day symptom recall period, ensuring minimal recall bias while accurately reflecting recent symptom burden. Convergent validity of Br-CUSP was demonstrated by inverse correlation with SF-12. Discriminant validity scores were significantly higher in all Br-CUSP domains among patients with ureteral stones compared to controls, with large effect sizes.

Quality of life should be recognized as a core outcome metric in the management of urolithiasis, providing insights beyond traditional endpoints such as SFR and complications (1, 20-24). Although Short Form 36, a generic questionnaire, is commonly used for assessing health-related quality of life in many medical conditions, it is not accurate enough to monitor quality of life in urinary stone disease (25).

The Wisconsin Stone Quality of Life (WISQOL) questionnaire is well-established PROM for nephrolithiasis (26). While WISQOL assesses broader urinary stone disease burden, CUSP focuses uniquely on the acute symptomatology of ureteral stones, offering more specific insights. Unlike WISQOL, which evaluates long-term QoL impact, CUSP captures recent (previous seven days) symptom burden, making it particularly useful for monitoring acute treatment effects (17). Future studies using Br-CUSP and WIQOL may help define their respective roles and determine whether Br-CUSP can serve as a complementary or superior alternative in the acute setting.

A key strength of our study lies in its rigorous and comprehensive validation methodology, which includes CFA, McDonald's omega for internal consistency, and robust construct validity testing. These methodological enhancements provide a level of psychometric rigor that extends beyond the original CUSP validation study (17). Notably, the inclusion of McDonald's omega offers a more reliable estimate of internal consistency than Cronbach's alpha alone. Furthermore, our validation was conducted in a demographically diverse population, supporting the broader applicability and generalizability of the CUSP questionnaire in varied clinical settings.

Nonetheless, this study is not without limitations. It was conducted at a single center, and a longitudinal responsiveness to treatment interventions was not assessed. Future research should explore Br-CUSP sensitivity to clinical changes over time and its correlations with objective clinical outcomes, such as SFR and complication rates.

## CONCLUSIONS

The Brazilian Cambridge Ureteral Stone Patient-Reported Outcome Measure is the first validated, disease-specific Patient Related Outcome Measure for ureteral stones in Brazilian Portuguese, addressing a crucial gap in patient-centered outcome assessment. Its strong psychometric properties make it a reliable tool for evaluating the acute impact of ureteral stones on quality of life. Future research should explore its application in clinical decision-making, particularly by correlating quality of life outcomes with stone-free rates and complication rates.

## Data Availability

All data generated or analysed during this study are included in this published article
