# Supplementary material for: Quality of Life in Patients with Ureteral Stones: Translation and Validation of the Brazilian Version of the Cambridge Ureteral Stone PROM (Br-CUSP)
Source: Int Braz J Urol. 2025 Nov 30;52(2):e20250551. doi: 10.1590/S1677-5538.IBJU.2024.0551 (PMC13124162; doi:10.1590/S1677-5538.IBJU.2024.0551)
Supplement: Supplementary file 1 [file 1677-6119-ibju-52-02-e20250551-Suppl1.pdf]

APPENDIX

Supplementary material 1 - Latent Variable, Indicators, and Their Respective Estimated Loadings for the Br-CUSP Scale.

Latent variables	Indicator	Standardized Factor Loading	Standard Error	z	p	R^2
**Factor 1**						
	#1	0.949	0.013	80.71	<0.001	0.901
	#2	0.953	0.013	74.968	<0.001	0.908
	#3	0.963	0.013	78.464	<0.001	0.927
	#4	0.935	0.015	66.646	<0.001	0.875
	#5	0.954	0.015	68.488	<0.001	0.910
	#6	0.970	0.014	74.086	<0.001	0.941
	#7	0.946	0.015	66.385	<0.001	0.895
	#8	0.927	0.020	49.318	<0.001	0.860
**Factor 2**						
	#9	0.943	0.01	70.35	<0.001	0.889
	#10	0.927	0.021	47.155	<0.001	0.860
	#11	0.924	0.023	42.789	<0.001	0.854
	#12	0.949	0.018	55.435	<0.001	0.900
	#13	0.956	0.019	52.475	<0.001	0.913
**Factor 3**						
	#14	0.934	0.020	60.19	<0.001	0.872
	#15	0.952	0.021	47.630	<0.001	0.906
	#16	0.962	0.024	43.063	<0.001	0.925
**Factor 4**						
	#17	0.935	0.020	50.54	<0.001	0.874
	#18	0.756	0.044	18.298	<0.001	0.572
	#19	0.880	0.032	29.783	<0.001	0.774
	#20	0.918	0.029	34.425	<0.001	0.843
**Factor 5**						
	#21	0.859	0.03	25.18	<0.001	0.738
	#22	0.904	0.062	17.071	<0.001	0.818
	#23	0.903	0.051	20.473	<0.001	0.816
**Factor 6**						
	#24	0.838	0.07	12.67	<0.001	0.702
	#25	0.622	0.117	6.363	<0.001	0.387
	#26	0.787	0.097	9.674	<0.001	0.620

Br-CUSP = Brazilian version of the Cambridge Ureteral Stone Patient-reported Outcome Measure

Supplementary material 2 - Spearman's Correlation Coefficients between Patients' Mean Scores in Each Domain and between Patients' Scores in Each Domain of the Br-CUSP and SF-12 Scale Dimensions.

Domain		Domain	*Rho*	Br-CUSP	SF-12	rho
Pain	-	Fatigue	0.82*	Total score	PCS	-0.67*
Pain	-	Work	0.81*	Total score	MCS	-0.64*
Pain	-	Sleep	0.73*	Pain	PCS	-0.65*
Pain	-	Anxiety	0.60*	Pain	MCS	-0.52*
Pain	-	Urinary symptoms	0.55*	Fatigue	PCS	-0.57*
Fatigue	-	Work	0.76*	Fatigue	MCS	-0.58*
Fatigue	-	Sleep	0.72*	Work	PCS	-0.68*
Fatigue	-	Anxiety	0.63*	Work	MCS	-0.50*
Fatigue	-	Urinary symptoms	0.50*	Sleep	PCS	-0.53*
Work	-	Sleep	0.66*	Sleep	MCS	-0.55*
Work	-	Anxiety	0.62*	Anxiety	PCS	-0.47*
Work	-	Urinary symptoms	0.51*	Anxiety	MCS	-0.66*
Sleep	-	Anxiety	0.63*	Urinary symptoms	PCS	-0.42*
Sleep	-	Urinary symptoms	0.52*	Urinary symptoms	MCS	-0.44*
Anxiety	-	Urinary symptoms	0.45*			

*p<0.001

Rho = spearman correlation; Br-CUSP = Brazilian version of the Cambridge Ureteral Stone Patient-reported Outcome Measure; SF-12 = Short-Form 12; PCS = Physical Component Score; MCS = Mental Component Score
